# Comparison of the Microstructural, Mechanical and Corrosion Resistance Properties of Ti6Al4V Samples Manufactured by LENS and Subjected to Various Heat Treatments

**DOI:** 10.3390/ma17051166

**Published:** 2024-03-01

**Authors:** Anna Antolak-Dudka, Tomasz Czujko, Tomasz Durejko, Wojciech J. Stępniowski, Michał Ziętala, Justyna Łukasiewicz

**Affiliations:** 1 Institute of Materials Science and Engineering, Military University of Technology, Kaliskiego 2, 00-908 Warsaw, Poland; tomasz.durejko@wat.edu.pl (T.D.); wojciech.stepniowski@wat.edu.pl (W.J.S.); justyna.lukasiewicz@wat.edu.pl (J.Ł.); 2 Multidisciplinary Research Center, Cardinal Stefan Wyszynski University in Warsaw, Marii Konopnickiej 1, 05-092 Dziekanów Leśny, Poland; m.zietala@uksw.edu.pl

**Keywords:** additive manufacturing, Ti6Al4V alloy, laser engineered net shaping, LENS, hot isostatic pressing, HIP

## Abstract

In this paper, the influences of two post-heat treatments on the structural, mechanical and corrosion resistance properties of additively manufactured Ti6Al4V alloys were discussed in detail. The materials were produced using the laser engineering net shaping (LENS) technique, and they were subjected to annealing without pressure and hot isostatic pressing (HIP) under a pressure of 300 MPa for 30 min at temperatures of 950 °C and 1050 °C. Annealing without pressure led to the formation of a thin plate structure, which was accompanied by decreasing mechanical properties and increasing elongation and corrosion resistance values. For the HIP process, the formation of a thick plate structure could be observed, resulting in the material exhibiting optimal mechanical properties and unusually high elongation. The best mechanical and corrosion resistance properties were obtained for the material subjected to HIP at 950 °C.

## 1. Introduction

Currently, additive manufacturing (AM) techniques are among the most dynamic and rapidly developing methods for the production of detailed components with complex geometries. Several AM techniques can be distinguished based on the batch material (polymers or metal alloys) used [1,2].

A quite popular additive manufacturing technique is laser engineering net shaping (LENS). LENS was developed and commercialized in 1997 by Optomec of Sandia National Laboratories [3,4]. With this technique, it is possible to reduce time and costs in the production of elements by significantly shortening the entire process, from design to manufacturing. The components manufactured from LENS devices are composed of metallic powder that is supplied directly into a location where a high-power laser beam is focused under a protective atmosphere of argon gas. In a layer-by-layer manner, near-net-shaped components are created with a dimensional accuracy comparably to the CAD model. All of the parameters, such as the amount of fed powder, power of the laser, feed rate of the working table and thickness of the layer, are precisely defined and fully controlled throughout the process [5]. The influences of these parameters on the mechanical properties and microstructures have been investigated [5,6,7]. These advantages result in a wide variety of applications, from regeneration of damaged parts to small batch manufacturing of personalized components.

There is a very wide range of batch materials available for use in LENS devices, and the most commonly used options are commercial engineering materials, such as steels, nickel-based super alloys and titanium and its alloys. A distinctive result of using the LENS technique to produce these materials is often the formation of nonequilibrium structures or additional phases that are impossible to obtain by conventional methods, which in turn leads to the generation of unique properties. This distinction arises due to the high cooling rate in the molten pool during the manufacturing process. A cooling rate of approximately 7 × 10^4^ K/s in the direct energy deposition (DED) process, which encompasses the LENS technique, was reported by Qian et al. [8].

Very popular materials of interest to researchers working in the field of AM techniques are titanium and its alloys. The alloy tested in this study is a lightweight commercial Ti6Al4V titanium alloy that has a number of very beneficial properties, such as a high specific strength, high melting point and low density. In addition, the very good corrosion resistance and biocompatibility of this alloy allow it to be widely used in the field of medical implants [9,10,11]. All of these advantages have led to its application in the medical and aerospace industry. Pure titanium has two allotropic varieties: low-temperature Ti-α crystals in a hexagonal close-packed (HCP) system and high-temperature Ti-β crystals in a body-centered cubic (BCC) system. The chemical composition of the Ti6Al4V alloy includes alloying elements—aluminum and vanadium—which stabilize the apparent α and β phases. For this reason, the structure of the alloy at room temperature consists of an α + β mixture. The nature of a two-phase structure can be very different from that of an equiaxed structure, ranging from bimodal to lamellar. The sizes, distributions and morphologies of these two phases strongly depend on the cooling rates during manufacturing [5,12]. According to a diagram illustrating the dependence of the phase transformations on the cooling rate for Ti6Al4V alloys [13], if the cooling rate is sufficiently high (>410 °C/s), the β phase transforms through a diffusionless process into a martensitic α’ phase. The formation of the α’ phase within long columnar prior β grains occurs primarily during the AM-based production of components composed of the Ti6Al4V alloy [14]. This formed α’ phase is the result of the fulfillment of the second condition leading to this result, which states that the temperature during the building of the elements must be higher than the martensitic transformation temperature (Ms). The temperature value depends on the amount and type of impurities present in the chemical composition of the alloy, and it ranges from approximately 575 °C to 800 °C [15,16]. The presence of the martensitic phase in the Ti6Al4V alloy plays a major role in the strength parameters. The yield point or tensile strength increases as both the elongation and plasticity decrease [17,18]. Galarraga et al. [17] showed that the presence of α’ needles increases the hardness of the material. 

To improve the ductility, postprocessing treatment is necessary for the Ti6Al4V alloy-based components produced by AM techniques. The most popular postprocessing technique is heat treatment [19]. This treatment mainly results in structural changes that alter the material properties. For the Ti6Al4V alloy, according to a pseudobinary phase diagram, the α→β transition temperature is approximately 980 °C [20]; therefore, for the decomposition of the α’ phase to occur during heat treatment, the process should be carried out at a similar temperature to that mentioned above. Yuan et al. [21] investigated the heat treatment effect (annealing at three different temperatures: 750 °C, 850 °C and 950 °C) on the compressive fatigue properties of titanium samples manufactured by the EBM method. By comparing the obtained results, the scholars found that annealing at 950 °C leads to the formation of a two-phase structure and significantly improves the ductility and fatigue strength. Kim et al. [22] studied Ti6Al4V samples produced by the SLM method and reported that samples subjected to heat treatment at 1040 °C for 1 h exhibit a lower steady-state creep rate than the as-built specimens. Moreover, the impacts of postprocessing heat treatment on the corrosion resistance and biocompatibility of the Ti6Al4V alloy are interesting. Reportedly, annealing the samples produced from the alloy at a temperature of 800 °C for 2 h significantly improves their corrosion resistance [23]. Conversely, heat treatment at a temperature of 820 °C for 4 h for SLM-based samples improves human bone mesenchymal stem cell (hBMSC) adhesion and proliferation [24].

Another example of a postprocessing heat treatment is hot isostatic pressing (HIP), where a high annealing temperature and pressure are applied simultaneously. This technique is used mainly for powder materials, and some related research has been conducted for Ti6Al4V alloys [25,26,27,28]. However, there is another route involving the use of the HIP technique as a postprocessing treatment for bulk samples produced by AM. Qiu et al. [29] studied the effects of using HIP (920 °C/103 MPa/4 h) on the changes in the porosity and microstructure that may occur during an experiment in SLM-based samples composed of Ti6Al4V powder. HIP is confirmed to completely transform martensite into a two-phase α + β structure and to close almost all of the pores created during sample manufacturing. Both the phenomena described above and their impacts on increasing the plasticity and fatigue endurance ratio parameters of the obtained materials have been described in previous papers [30,31,32]. The structural changes occurring in the Ti6Al4V alloy presented in the literature mainly concern the SLM process. Given the significant sensitivity of this material to technological history, it is necessary to investigate the changes occurring during the additive manufacturing process and subsequent thermal treatment.

In a recent paper, the authors attempted to comprehensively analyze results obtained from the use of two postprocessing heat treatments. The treatments—classic annealing and hot isostatic pressing—were carried out at two different temperatures. The researchers bore in mind that the use of a postprocessing heat treatment is necessary for samples produced with the Ti6Al4V alloy by AM techniques due to the microstructure formed during the manufacturing process. The mechanical properties were compared—microhardness, corrosion resistance and, primarily, the newly created microstructure α + β, which naturally has a large impact on the above properties.

## 2. Materials and Methods

### 2.1. Manufacturing of the Samples

The Ti6Al4V samples were manufactured using an Optomec LENS MR-7 device (Albuquerque, NM, USA). The scheme of the laser engineered net shaping system has been described in other papers [33,34]. The process of sample production by this method consisted of the layer-by-layer building of the elements previously designed by a CAD program from metallic powders, which were melted by a 500-W IPG YLR-500 fiber laser (IPG Photonics Corporation, Oxford, MS, USA). The working chamber was filled with an ultraclean argon atmosphere, and the oxygen content was controlled inside. For research purposes, cylindrical samples with dimensions of 10 mm × 100 mm (diameter × height) were built on a 7-millimeter-thick Ti6Al4V substrate plate, which was sandblasted and cleaned with acetone. The zig-zag printing strategy for a single layer was used. Each of the built layers was rotated by 30 degrees compared to the previous one without any dwell time between them. The manufactured cylinders were removed from the substrate using a BP-97d electrodischarge machine (ZAP-BP, Kutno, Poland). The parameters of the manufacturing process, such as the laser power and powder flow rate or oxygen content, ensured good metallurgical and dimensional quality of the produced samples. These parameters are presented in Table 1.

**Figure 1:**
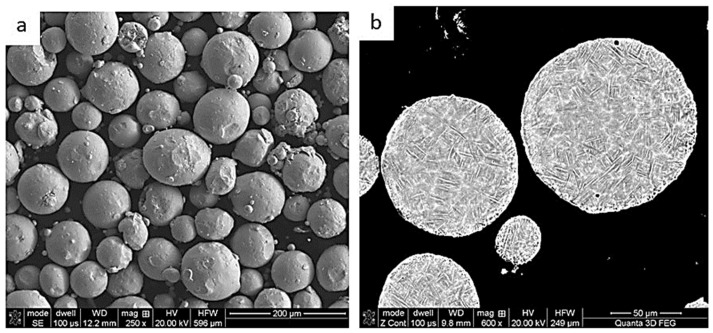
SEM micrographs of the morphology (**a**) and cross-section (**b**) of the Ti6Al4V batch powder.

The batch material used for building the cylindrical samples was Ti6Al4V powder supplied by the TLS Technik GmBH & Co Company (Bitterfeld-Wolfen, Germany). The chemical composition of the powder was confirmed by a certificate provided by the above company, and the content of individual elements are shown in Table 2. This powder was made using an argon atomization method, which determined the spherical shape of the particles, and the sizes of the particles ranged from 45 to 105 µm. These findings were confirmed by scanning electron microscopy (SEM) observations (Figure 1a), which revealed that the surfaces of the particles were mostly smooth, and that small particles were attached to large particles. The cross-sectional micrograph (Figure 1b) revealed that some small pores were present inside the particles.

To observe the structural changes occurring in the material, the cylindrical samples produced by the LENS technique were subjected to two types of postprocessing treatments (Table 3).

Annealing after processing was conducted at 950 °C and at 1050 °C for 30 min for both treatments. The process was conducted in a Nabertherm R80/b750/12-B170 tubular furnace (Nabertherm GmbH, Lilienthal, Germany) at low vacuum (×10^−2^ mbar), and before heating, the tube was purged with argon. The samples were cooled in the furnace. The conditions of the postprocessing heat treatment were selected based on our experience and on the available literature [20,35,36,37].

The second type of postprocessing heat treatment of the samples manufactured by the LENS technique was hot isostatic pressing (HIP). The temperature and duration of the HIP process were the same as those used for the pressure-free heat treatment, i.e., 950 °C for 30 min and 1050 °C for 30 min. The pressure in the working chamber was 300 MPa, and a protective pure argon (99.999%) atmosphere was applied. The specimens were cooled in the HIP chamber.

For comparison, a Ti6Al4V reference sample was taken from a commercially manufactured hip joint endoprosthesis.

### 2.2. Research Methods

Samples obtained during the LENS process and after two different postprocessing heat treatments were subjected to various material testing methods that are commonly used in materials engineering, in order to characterize the microstructure, mechanical properties and corrosion resistance of the manufactured materials. 

**Figure 2:**
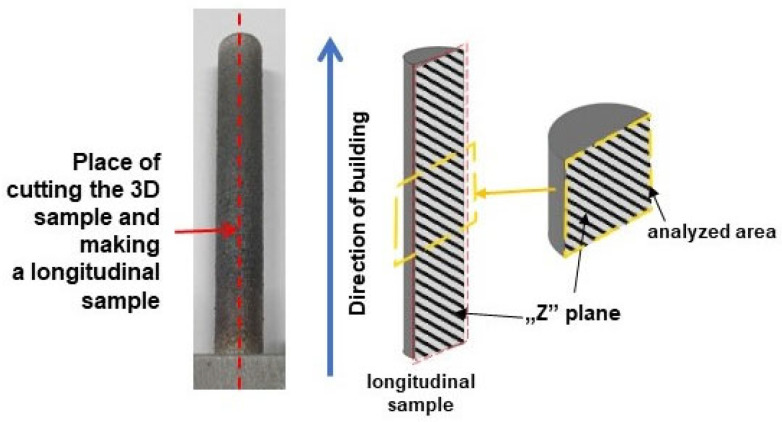
Diagram of metallographic sampling in the “z” plane parallel to the deposition direction; the longitudinal sample and the place of taking it was marked the red dashed line, the analyzed area was marked the dashed yellow line.

The specimens for metallographic observation were taken from the centers of cylindrical samples in the “z” plane parallel to the deposition direction during the LENS process (Figure 2). Afterward, the samples were mounted in thermosetting resin, ground with 600-mesh, 1200-mesh and 2400-mesh grinding papers, and their surfaces were chemically polished using MD-Chem discs with 0.25% colloidal silica in conjunction with 30% hydrogen peroxide. To reveal their microstructures, the specimens were etched with Kroll’s reagent.

The samples were prepared in this manner and subjected to microscopic observation. A Nikon (Tokyo, Japan) MA-200 optical microscope was used for macrostructural and microstructural observations. Additionally, the microstructures, fracture surfaces, chemical compositions and quantitative phases were investigated using an FEI Quanta 3D (Eindhoven, Netherlands) dual-beam field emission gun scanning electron microscope equipped with energy dispersive spectroscopy (EDS) and electron backscatter diffraction (EBSD).

The total porosity fraction in the full volume of each specimen taken from the center of cylindrical samples was determined using a Nikon MA-200 optical microscope equipped with an NIS-Elements BR 3.8 computer image analyzer. The degree of porosity for each sample was determined as the ratio of the measured pore area to the entire observed area. The analysis was carried out in both directions, taking into account statistically selected regions observed at magnification 100×, which resulted in thirty examined areas. The mean values and standard deviations were calculated for all obtained porosity results of each specimen.

**Figure 3:**
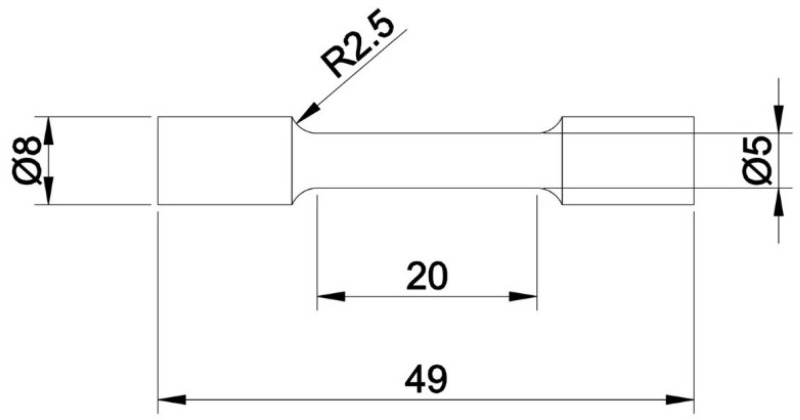
Dimensions of the samples used in the tensile tests (all dimensions are in mm).

The mechanical properties of the samples manufactured by the LENS technique directly after processing and after pressure-free and HIP heat treatments were identified using an INSTRON (Norwood, MA, USA) 8862 tensile testing machine with extensometer 2630-107 at a crosshead speed of 1 mm/min according to ISO 6892–1:2019 [38]. The tensile tests were performed on three identical samples under each condition. The dimensions of the tensile samples are shown in Figure 3. The yield point, tensile strength and elongation were estimated from the original stress-strain curves.

The Vickers microhardness measurements were performed using a Shimadzu microhardness tester under a load of 100 g for 10 s. The average microhardness values were calculated from at least 10 indentations, and the standard deviation was determined.

The phase compositions of the specimens were studied via X-ray diffraction (XRD) using a Seifert 3003 TT diffractometer (Rich. Seifert & CO. GmbH, Ahrensburg, Germany) with Cu K_α_ radiation (α = 1.5418 Å). The 2θ range was 20–120 degrees with a step of 0.02 deg and an exposure time of 3 s.

All of the electrochemical experiments were performed with a 0531 Atlas-Sollich potentiostat (Atlas-Sollich Z.S.E., Gdańsk, Poland). The experiments were performed in an electrochemical cell containing 100 mL of 0.9% NaCl, where the investigated samples were working electrodes, a platinum plate was the counter electrode and Ag|AgCl|sat. KCl was used as the reference electrode. The surface area of the investigated samples was limited by the rubber O-ring seal to 0.196 cm^2^. Prior to polarization, the open cell potential (OCP) was recorded for 1 h. Immediately after this measurement, a linear potential scan was performed at a 1 mV/s rate, which started 100 mV below the last OCP value. The polarization curves were recorded to 1.2 V vs. Ag|AgCl. The corrosion potential and corrosion current density were estimated using Tafel plots. Each experiment was performed three times.

## 3. Results and Discussion

### 3.1. Influences of Various Heat Treatments on the Porosities, Microstructures and Phase Compositions of Ti6Al4V Alloys Manufactured Using LENS

**Figure 4:**
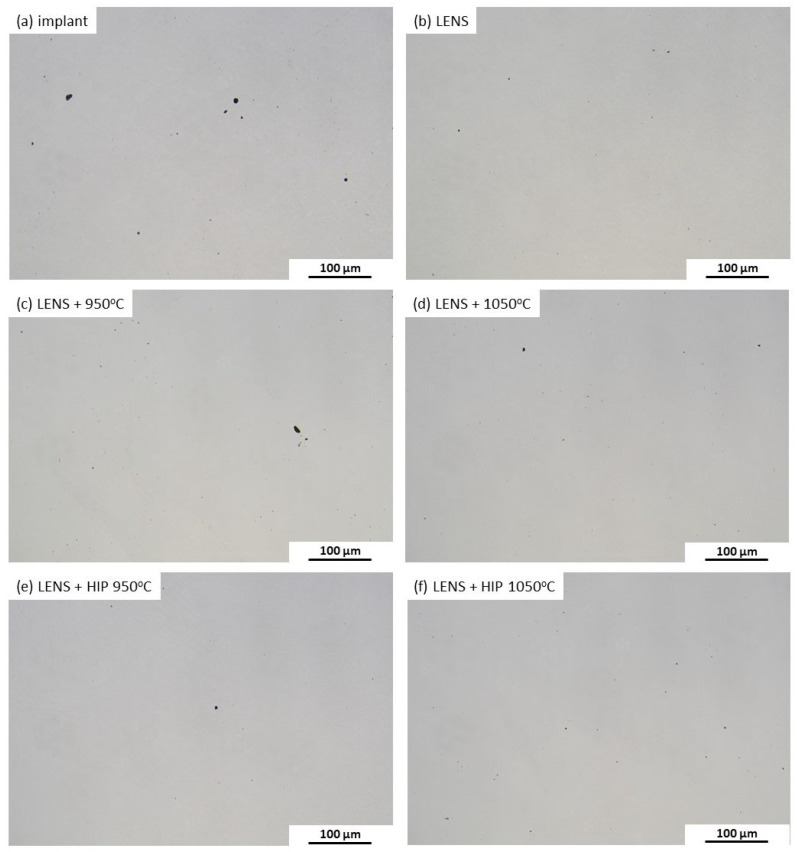
Micrographs revealing the porosities of a commercial hip joint implant (**a**) and samples manufactured by the LENS technique directly after processing (**b**) and annealing for 30 min at 950 °C (**c**) and 1050 °C (**d**) or HIP for 30 min at 950 °C (**e**) and 1050 °C (**f**).

The optical microscopy observations (Figure 4) and porosity measurements (Table 4) show that neither annealing nor HIP substantially affected the porosities of the samples relative to the original material directly after LENS manufacturing. Notably, the porosity of the AM-based material (0.08%) is lower than that of a commercially available hip joint implant (0.18%). Slight changes in the tested samples after different processing times are noticeable, but it can be reasonably assumed that they are comparable within the measurement error. The low porosities of the additively manufactured materials prove the high quality of the input material (no primary porosity) and the accurate selection of manufacturing process parameters. Ensuring the high metallurgical quality of additively manufactured materials suggests that further heat treatment, both by annealing and HIP, does not have a significant impact on the final porosity. This finding indicates that any differences in mechanical properties only result from structural differences and not from the degree of porosity of the material.

**Figure 5:**
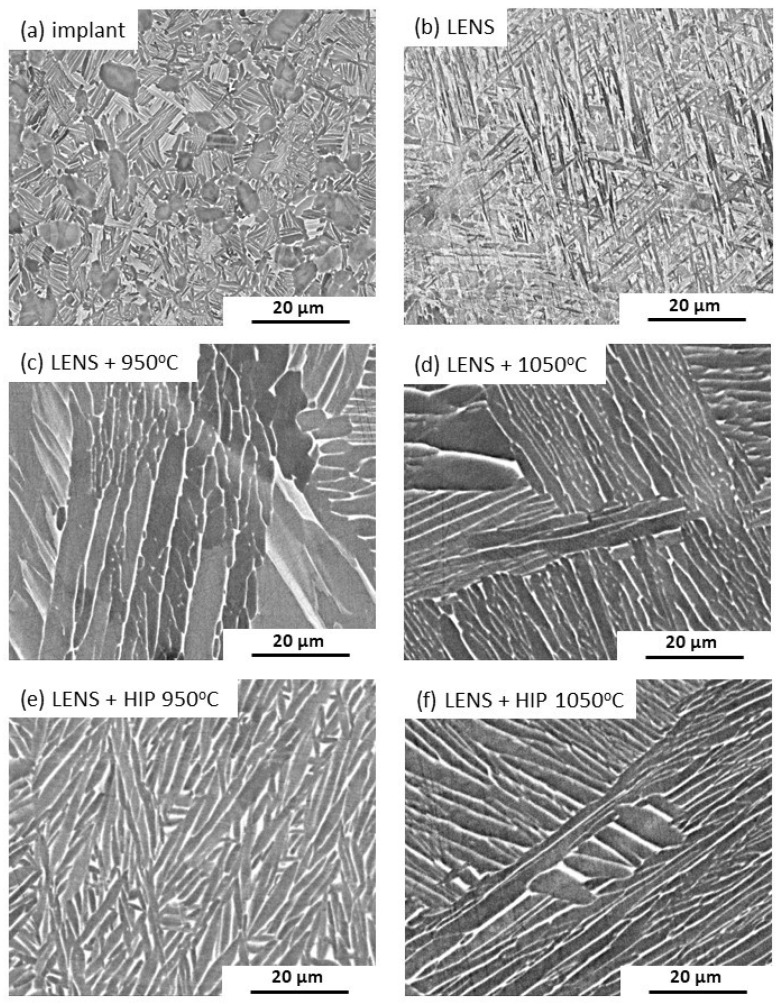
SEM micrographs revealing the microstructures of commercial hip joint implants (**a**) and samples manufactured by the LENS technique directly after processing (**b**) and annealing for 30 min at 950 °C (**c**) and 1050 °C (**d**) or HIP for 30 min at 950 °C (**e**) and 1050 °C (**f**).

Commonly used implants made of Ti6Al4V alloy are characterized by a two-phase lamellar α + β structure, ensuring good mechanical properties, such as a yield strength of 795 MPa, an ultimate tensile strength of 860 MPa and an elongation of 10% (Figure 5a) [39]. The structure of the material produced using the LENS technique (Figure 5b) is typical for additively manufactured titanium alloys, and is characterized by the martensitic structure of the α’ phase [13,14,15,16,17].

Annealing at 950 °C for 30 min leads to the transformation of the α’ martensitic structure into a plate-shaped α + β structure (Figure 5c) with a lower beta phase content than that of a commercial implant. An increase in the heating temperature to 1050 °C leads to significant growth of the structure, which may ultimately result in a decrease in the plasticity of the material [40]. Unlike pressure-free annealing, HIP leads to the formation of a highly fragmented plate shape structure. As in the case of pressure-free annealing, an increase in the temperature during HIP leads to noticeable plate growth.

**Figure 6:**
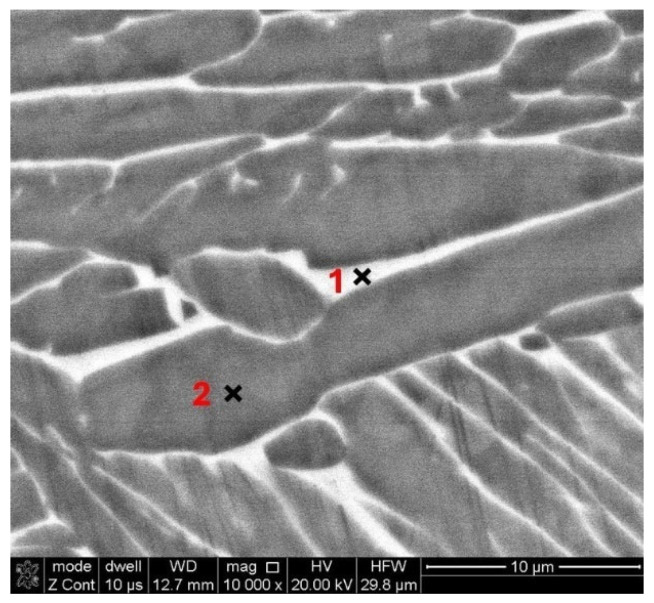
SEM sample micrograph (LENS + 950 °C) with marked areas where the EDS investigations occurred.

To identify the phase structures of the light and gray areas shown in Figure 5, an analysis of the chemical compositions of the microareas is performed. The chosen analysis site for a sample is shown in Figure 6.

The results of the quantitative analysis of the microareas for samples with different technological histories are presented in Table 5. Based on the contents of the α and β stabilizing elements, the light areas marked in Figure 6 as “1” are clearly the β phase with an increased content of vanadium and a reduced content of aluminum [41]. However, the gray areas (“2” in Figure 6) are created by the alpha phase with increased aluminum and reduced vanadium concentrations [41].

**Figure 7:**
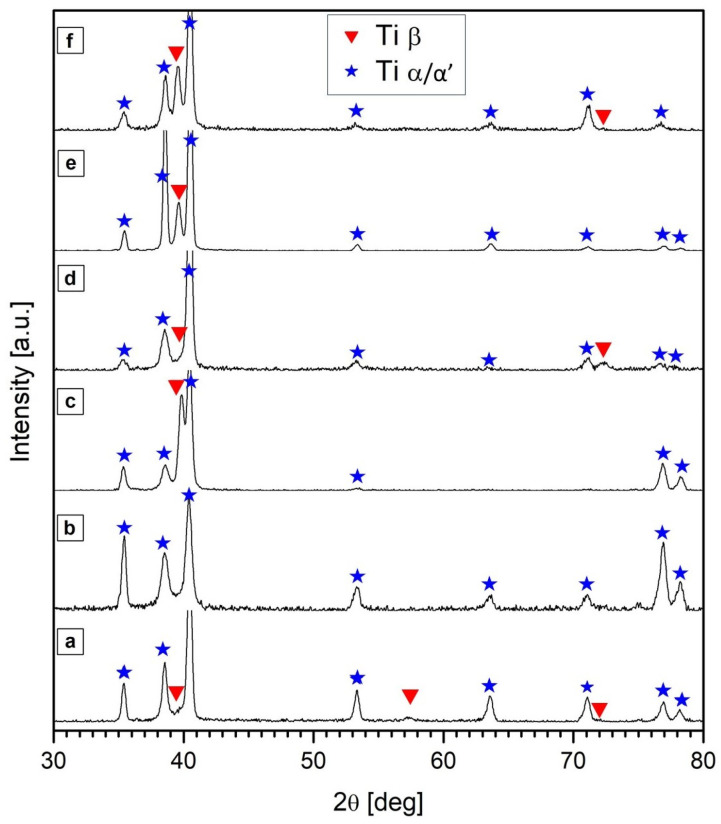
Sequences of XRD patterns for commercial hip joint implants (**a**) and samples manufactured by the LENS technique directly after processing (**b**) and annealing for 30 min at 950 °C (**c**) and 1050 °C (**d**) or HIP for 30 min at 950 °C (**e**) and 1050 °C (**f**).

Our microstructural observations were confirmed by XRD (Figure 7), where the XRD pattern for the commercial implant is characterized by the presence of peaks originating from the α and β phases. For additively manufactured materials, only o peaks originating from the α’ phases are observed, which is typical for additively manufactured Ti6Al4V alloys [13,14,15,16,17,42,43]. For the remaining samples, both annealed and HIP-processed, peaks originating from the α and β phases can be observed.

**Figure 8:**
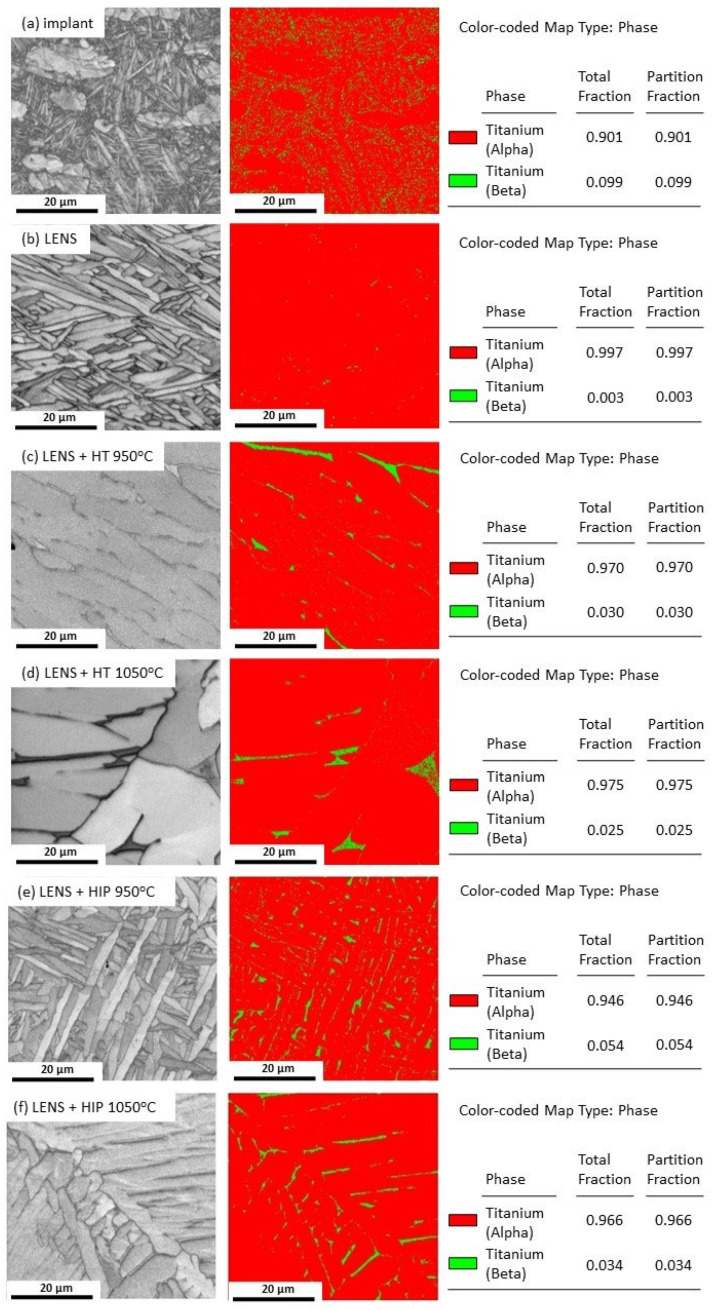
EBSD phase distribution maps of commercial hip joint implants (**a**) and samples manufactured by the LENS technique directly after processing (**b**) and annealing for 30 min at 950 °C (**c**) and 1050 °C (**d**) or HIP for 30 min at 950 °C (**e**) and 1050 °C (**f**).

Quantitative analysis of the beta-phase volume fraction was performed using EBSD. The test results are presented in Figure 8 and Table 6. The α phase and β phase are colored red and green, respectively, in the EBSD maps. The test results confirm that the volume fraction of the β phase that is typical for implants composed of the Ti6Al4V alloy is approximately 10%. A negligibly low content of the β phase (0.3%) is typical for the AM-based Ti6Al4V material, and it results from the extremely high cooling rate accompanying this process [42]. However, pressure-free annealing at 950 °C and 1050 °C increases the volume fractions of the β phases to 3.0% and 2.5%, respectively. The highest fraction of the β phase (5.4%) can be observed for the material after HIP at 950 °C. An increase in the process temperature to 1050 °C causes the grains to grow and a 3.4% decrease in the volume fraction of the β phase, which may affect the mechanical properties of the material.

Ganor et al. [43] reported that as-built samples have the lowest β phase contents (less than 1%). However, contrary to the results obtained in our studies, the scholars observed an increase in the fraction of the β phase with an increase in the temperature during the HIP process, reaching 7% at 1100 °C. This difference may be related to the different technological history of both materials—different manufacturing techniques and conditions of the HIP process.

### 3.2. Influences of Various Heat Treatments on the Mechanical Properties and Fracture Behaviors of Ti6Al4V Alloys Manufactured Using LENS

The test results presented in Table 7 indicate that the material immediately after conducting the LENS process has the highest microhardness (356 ± 12 HV0.1). Considering its martensitic structure and the supersaturation of the α phase with vanadium, this phenomenon has been widely understood and described [13,14,15,16,17,42,43]. Several researchers have reported even higher hardness values for Ti6Al4V alloys immediately after the additive manufacturing process, e.g., 372 ± 4 HV0.1 [44] and 412 ± 23 HV0.3 [45]. Importantly, the microhardness of the material after the LENS process is only slightly greater than the microhardness of a commercially available implant made of the Ti6Al4V alloy, which is 342 ± 6 HV0.1 Annealing the AM material at temperatures of 950 °C and 1050 °C causes noticeable softening and a decrease in the microhardness to 329 ± 10 HV0.1 and 322 ± 18 HV0.1, respectively. Notably, the HIP process carried out at analogous temperatures leads to the creation of a material with very similar hardness values of 325 ± 7 HV 0.1 and 320 ± 12 HV 0.1, respectively.

**Figure 9:**
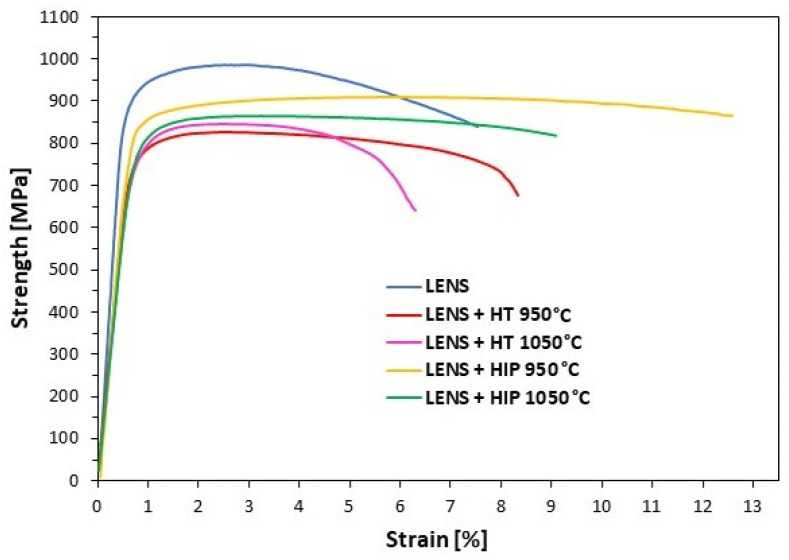
Tensile test curves for samples manufactured by the LENS technique directly after processing, after postprocessing heat treatment or after HIP.

The results of the static tensile test (Table 8) show that the highest yield strength (YS; 729 ± 32 MPa) and ultimate tensile strength (UTS; 995 ± 8 MPa) are demonstrated by the material immediately after production using LENS. Compared to the reference implant material, the additively manufactured material is characterized by increased strength and slightly reduced plasticity. Heat treatment involving pressure-free annealing decreases the strength to a value typical for commercial implants. When annealed at 950 °C, the plasticity increases to 7.9 ± 0.9%. Unfortunately, the increase in the heating temperature and the accompanying growth of plate-shaped grains decreases the elongation to the level of the AM-based material (6.6 ± 1.0%). Exemplary tensile test curves of samples manufactured by the LENS technique and subjected to various heat treatments are shown in Figure 9.

The best mechanical properties (high yield strength, ultimate tensile strength and elongation) are obtained for the material subjected to HIP at 950 °C. Remarkably, the plasticity of the material after such heat treatment is greater than that of commercial implants. Moreover, an unusual simultaneous increase in strength and plasticity can be seen. This may be the result of the presence of characteristic lamellar structure, which is created as a result of heat treatment with the application of pressure. According to Wu et al. [46], such a structure can promote the uniformity of plastic flow by preventing strain localization. However, increasing the temperature during the HIP process to 1050 °C causes a slight decrease in the mechanical properties and elongation, which is caused by the growth of the plate shape structure.

### 3.3. Influences of Various Heat Treatments on the Corrosion Resistance of Ti6Al4V Alloys Manufactured by LENS

**Figure 10:**
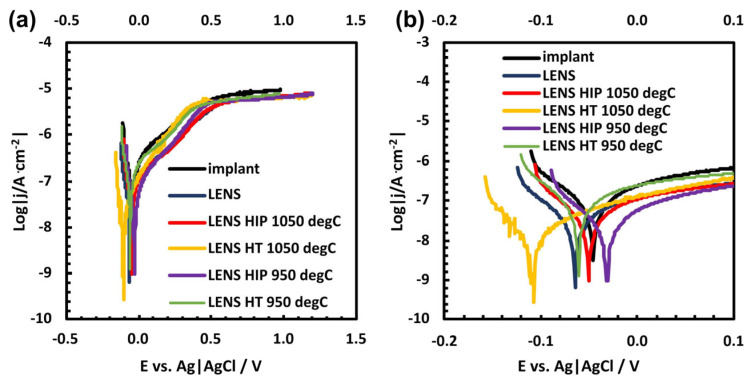
Exemplary polarization curves of the investigated materials recorded in 0.9% NaCl in the full range (**a**) and in the range where the corrosion potential and corrosion current density are distinguishable (**b**).

Exemplary polarization curves for all of the investigated materials are presented in Figure 10. A wide range of graphs reveals the passive range (Figure 10a) for all of the patients, similar to that of the reference hip joint implant. Importantly, there are distinct differences in the corrosion potential and current density (Figure 10b).

Table 9 shows the average values of the corrosion potential and corrosion current density. These data can lead to certain conclusions and be applied to plot the polarization curves. The lowest corrosion potential, translating into the greatest corrosion susceptibility (−104 mV vs. Ag|AgCl), was found for the reference implant. The same samples are characterized by the ones with the lowest corrosion current densities, corresponding to one of the lowest corrosion rates (98 nA/cm^2^). Samples fabricated by LENS without subsequent posttreatment are characterized by more than three cycles of current density.

However, both heat treatment and HIP improved the corrosion performance of the samples. Notably, after pressure-free heat treatment, the samples exhibited slightly better corrosion performance than the samples after HIP. After heat treatment, the samples showed greater average corrosion potential and slightly greater corrosion current densities than those recorded for the samples after HIP. Nevertheless, the results obtained for samples after heat treatment ranged widely: the corrosion potentials of samples after LENS and heat treatment at 1050 and 950 °C range from −131 to 105 mV vs. Ag|AgCl and from −88 to 127 mV vs. Ag|AgCl, respectively. Simultaneously, the polarization curve of the samples heat treated at 1050 °C in Figure 10 sufficiently depicts this trend. The corrosion potentials of the samples obtained by LENS and HIP treatment at 1050 and 950 °C range from −116 to 33 mV vs. Ag|AgCl and from −100 to −59 mV vs. Ag|AgCl, respectively. Moreover, the samples obtained via LENS followed by HIP at 1050 and 950 °C have the lowest corrosion current densities, namely, 140 and 77 nA·cm^−2^, respectively (Table 9). Table 9 unambiguously shows that first conducting LENS and then performing HIP yields the samples with the best corrosion performance. Moreover, the repetitiveness of the corrosion parameters of the samples formed by LENS followed by HIP makes this combination reliable.

## 4. Conclusions

In this research, we appropriately selected the technological parameters for additive manufacturing to ensure that a Ti6Al4V alloy with a negligible volume fraction of porosity and satisfactory mechanical properties was obtained. The postprocessing procedure involving pressure-free heating at 950 °C and 1050 °C for 30 min reconstructed the martensitic structure into a thick plate structure. This phenomenon was accompanied by a decrease in the mechanical properties (YS = 782 MPa, UTS = 838 MPa) and increases in the plasticity (A = 7.9%) and the corrosion resistance.

HIP at a temperature of 950 °C led to optimal mechanical properties, elongations exceeding that of commercially available implants, and high corrosion resistance. This treatment is recommended for AM implant components composed of the Ti6Al4V alloy. An increase in the temperature during the HIP process to 1050 °C deteriorated the plasticity of the material (A = 6.6%); thus, this procedure is not recommended.

## Figures and Tables

**Table 1:** Parameters of the manufacturing of cylindrical samples by the LENS technique.

Laser Power (W)	Powder Flow Rate (g/min)	Working Table Feed Rate (mm/s)	Single Layer Thickness (mm)	Batch Material	Substrate Material	Oxygen Content (ppm)
300	3.5	17	0.3	Ti6Al4V	Ti6Al4V	>5

**Table 2:** Chemical composition (at. %) of Ti6Al4V powder according to data provided by the TLS Technik GmBH & Co Company.

Ti (%)	Al (%)	V (%)	C (%)	Fe (%)	O (%)	N (%)	H (%)
bal.	5.5–6.75	3.5–4.5	max. 0.08	max. 0.40	max. 0.20	max. 0.05	max. 0.015

**Table 3:** Parameters of the postprocessing heat treatment and HIP processes conducted for samples obtained by the LENS technique.

Process	Temperature (°C)	Time (min)	Pressure (MPa)
LENS + postprocessing heat treatment	950 °C	30	10^−6^
	1050 °C	30	10^−6^
LENS + HIP	950 °C	30	300
	1050 °C	30	300

**Table 4:** Porosities of commercial hip joint implants and samples manufactured by the LENS technique after postprocessing heat treatment and HIP.

Process	Temperature (°C)	Porosity (%)
Implant	-	0.18 ± 0.05
LENS	-	0.08 ± 0.02
LENS + postprocessing heat treatment	950 °C	0.09 ± 0.01
	1050 °C	0.1 ± 0.01
LENS + HIP	950 °C	0.03 ± 0.01
	1050 °C	0.07 ± 0.02

**Table 5:** Content of each element (wt.%) measured via EDS.

Element	LENS		LENS + P-P HT				LENS + HIP			
			950 °C		1050 °C		950 °C		1050 °C	
	1	2	1	2	1	2	1	2	1	2
Ti	88.1	89.9	78.0	91.7	76.4	91.9	85.0	89.9	80.9	91.5
Al	6.2	6.4	2.7	6.9	2.6	7.0	4.6	7.7	4.0	7.1
V	5.7	3.8	19.3	1.5	21.0	1.1	10.4	2.4	15.1	1.4

**Table 6:** Volume fraction of the β phase estimated for EBSD phase maps of commercial hip joint implants and samples manufactured by LENS directly after processing, and then annealing or HIP for 30 min at 950 °C or 1050 °C.

Sample		β Phase Volume Fraction (%)
Implant	-	9.9
LENS	-	0.3
LENS + postprocessing heat treatment	950 °C	3.0
	1050 °C	2.5
LENS + HIP	950 °C	5.4
	1050 °C	3.4

**Table 7:** Microhardness HV0.1 (average ± std. dev.) measured for commercial hip joint implants and for samples manufactured by the LENS technique directly after processing and annealing or HIP for 30 min at 950 °C or 1050 °C.

Sample		Microhardness HV0.1
Implant	-	342 ± 6
LENS	-	356 ± 12
LENS + postprocessing heat treatment	950 °C	329 ± 10
	1050 °C	322 ± 18
LENS + HIP	950 °C	325 ± 7
	1050 °C	320 ± 12

**Table 8:** Average values ± std. dev. of the YS, UTS and El. determined from tensile tests conducted for samples manufactured by the LENS technique directly after processing, after postprocessing heat treatment or after HIP.

Process	Temperature (°C)	YS (MPa)	UTS (MPa)	El. (%)
Implant *	-	795	860	10
LENS	-	929 ± 32	995 ± 8	6.8 ± 1.7
LENS + P-P HT	950 °C	782 ± 18	838 ± 18	7.9 ± 0.9
	1050 °C	789 ± 18	840 ± 18	6.6 ± 1.0
LENS + HIP	950 °C	812 ± 36	904 ± 26	12.5 ± 3.1
	1050 °C	788 ± 7	860 ± 7	7.2 ± 2.3

* Data from the literature [39].

**Table 9:** Corrosion parameters of samples manufactured in different manners.

Process	Temperature (°C)	Corrosion Potential vs. Ag|AgCl/mV	Corrosion Current Density/ nA·cm^−2^
Implant		−104	98
LENS	-	−21	310
LENS + P-P HT	950 °C	−34	360
	1050 °C	−6	120
LENS + HIP	950 °C	−74	77
	1050 °C	−52	140

## Data Availability

The data presented in this study are available upon reasonable request from the corresponding authors.
